# The Use of Pepsin

**Published:** 1870-12

**Authors:** 


The Use of Pepsin.—Dr. E. P. Hurd ridicules the idea that pepsin can be of the value as an aid to digestion that many seem to regard it. Most of the substance of the pepsin we use is *starch*, and in any case, be it never so strong, a scruple of it can only digest eighty or ninety grains of aliment.

Boudault's pepsin only digests about fourtimes its own weight of albumen.

How far is our ordinary dose going to help a poor victim of indigestion through the solution of an ordinary meal?

He strongly suspects that with regard to the real effect of this agent we are very much deceived.—*Medical and Surgical Journal.*

